# Causal association between JAK2 and erectile dysfunction: a Mendelian randomization study

**DOI:** 10.1186/s12610-023-00192-0

**Published:** 2023-07-06

**Authors:** Yu-Jia Xi, Rui Wen, Ran Zhang, Qi-Rui Dong, He-yi Zhang, Qin-yi Su, Sheng-Xiao Zhang

**Affiliations:** 1 Department of Urology, Second Hospital of Shanxi Medical University, Shanxi Medical University, Taiyuan, P.R. China; 2 Shanxi Provincial Key Laboratory of Rheumatism Immune Microecology, Shanxi Province, Taiyuan, P.R. China; 3 Key Laboratory of Cellular Physiology at Shanxi Medical University, Ministry of Education, Shanxi Province, Taiyuan, P.R. China; 4 Department of Rheumatology, Second Hospital of Shanxi Medical University, Shanxi Medical University, 382 Wuyi Road, Taiyuan, Shanxi 030001 P.R. China

**Keywords:** Erectile dysfunction, JAK2, Mendelian randomization, Dysfonction érectile, JAK2, Randomisation mendélienne, Dysfonction érectile, JAK2, Randomisation mendélienne

## Abstract

**Background:**

As one of the most critical proteins in the JAK/STAT signaling pathway, Janus kinase 2 (JAK2) is involved in many biological processes and diseases. Several observational studies have reported the role of JAK2 in erectile dysfunction. However, the causal relationship between JAK2 and erectile dysfunction remains unclear. Here we investigated the causal relationship between JAK2 and erectile dysfunction.

**Results:**

Genetically predicted JAK2 was causally associated with erectile dysfunction in inverse variance weighting (OR = 1.109, 95% CI = 1.029–1.196, p = 0.007) and weighted median method (OR = 1.117, 95% CI = 1.003-1.245, p = 0.044). No heterogeneity was observed in Cochran Q-test (*p* = 0.855) and MR-PRESSO (*p* = 0.866). Pleiotropy was not observed in our study (*p* = 0.617).

**Conclusions:**

These findings highlighted JAK2 as a risk factor for erectile dysfunction and proved the causal relationship between JAK2 and erectile dysfunction, suggesting that targeting JAK2 signaling might be a novel and promising therapeutic candidate in the treatment of erectile dysfunction.

## Résumé

**Contexte:**

En tant que l’une des protéines les plus critiques de la voie de signalisation JAK/STAT, Janus kinase 2 (JAK2) est impliquée dans de nombreux processus biologiques et maladies. Plusieurs études observationnelles ont rapporté le rôle de JAK2 dans la dysfonction érectile. Cependant, la relation causale entre JAK2 et la dysfonction érectile reste incertaine. Ici, nous étudions la relation causale entre JAK2 et la dysfonction érectile.

**Résultats:**

JAK2 génétiquement prédit était causalement associée à la dysfonction érectile par pondération de variance inverse (OR = 1,109, IC à 95% = 1,029–1,196, *p* = 0,007) et la méthode médiane pondérée (OR = 1.117, IC à 95% = 1.003-1.245, *p *= 0.044). Aucune hétérogénéité n’a été observée dans le test Q de Cochran (*p* = 0,855) et MR-PRESSO (*p* = 0,866). La pléiotropie n’a pas été observée dans notre étude (*p* = 0,617).

**Conclusions:**

Ces résultats ont mis en évidence JAK2 comme un facteur de risque de dysfonction érectile et ont prouvé la relation causale entre JAK2 et la dysfonction érectile; ils suggèrent que le ciblage de la signalisation JAK2 pourrait être un candidat thérapeutique nouveau et prometteur dans le traitement de la dysfonction érectile.

## Background

Erectile dysfunction (ED) is a common male sexual dysfunction that refers to the persistent inability of the penis to achieve or maintain an erection sufficient for satisfactory sexual intercourse and mainly affects men over the age of 40 [1]. Patients with ED are often reluctant to discuss their hidden disorders in public because of the specificity of the disease. Moreover, ED brings great psychological and economic burdens to patients, which leads them to anxiety and even depression [2].

The protein encoded by the Janus kinase 2 (JAK2) is a non-receptor tyrosine kinase, a member of the Janus kinase family[3]. It contains a JAK homology pseudokinase (JH2) domain that regulates the adjacent protein kinase domain (JH1). The JAK signal transducer of activators of transcription (STAT) pathway (JAK/STAT) is now recognized as an evolutionarily conserved signaling pathway, which is involved in many crucial biological processes, including cell proliferation, differentiation, apoptosis, and immune regulation[3, 4]. Only two studies have reported the role of JAK2 in ED [5, 6]. But the causal relationship between JAK2 and ED was unclear.

Moreover, JAK/STAT inhibitors have been gradually used in the clinical treatment of male diseases and achieved good therapeutic effects improving the patients’ conditions. Hao Li et al. reported that down-regulate JAK2 expression rodents had an improvement in erectile function by improving the expression of the down-regulated NO/cGMP pathway[6]. However, these studies are confounded by mediating or confounding factors, which, together with bias, twisting, and reverse causation, limit the ability of observational pilot studies to identify causal relationships[7].

**Fig. 1:**
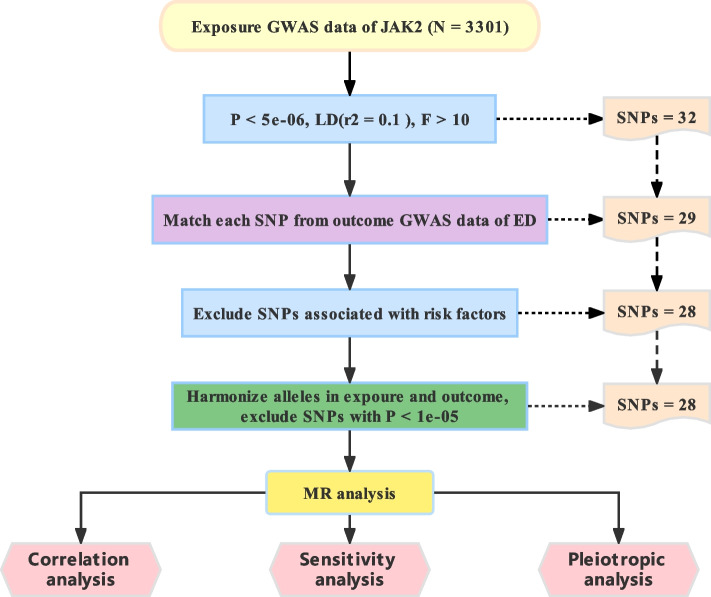
Flow chart of this study

Mendelian randomization (MR) analysis is based on the random assignment of parental alleles to offspring at the time of conception, with the help of instrumental variables to infer the relationship among genotype, intermediate phenotype, and disease outcome, and thus infer causality between exposure and outcome[2, 8]. Similar to randomized controlled trials (RCT), the random assignment of alleles is identical to a random grouping of samples in RCT and can correct for confounding factors that interfere with the results[9]. Accordingly, we applied publicly available genome-wide association studies (GWAS) databases to examine the causal associations between JAK2 (exposure factor) and ED (outcome factors) by a two-sample MR-controlled analysis in this study. The flow of this study is shown in Fig. 1.

## Methods

### GWAS data of JAK2 for instrumental variables

The data of tyrosine-protein kinase JAK2 (3,301 European samples, 10,534,735 SNPs) were collected as an exposure factor from a publicly available database (IEU OpenGWAS Project, https://gwas.mrcieu.ac.uk/) [10]. SNPs correlated with JAK2 were obtained from the IEU database. Under the threshold of *P* < 5 × 10^–6^ and pairwise *r*^2^ < 0.1. F statistics were used to evaluate the strength of the instrumental variables[11]. The threshold F < 10 defines the weak instrumental variable, so the deviation caused by the weak instrumental variable can be ignored.

### GWAS data of ED for outcomes

ED's GWAS summary data came from the latest R8 release of the FinnGen project (https://r8.finngen.fi/), which was also of European origin [12]. This ED data contained 20,151,730 SNPs of 1973 ED cases and 149,557 control samples.

### Elimination of confounding factors

We examined PhenoScanner (www.phenoscanner.medschl.cam.ac.uk) for potential confounders, including psychiatric factors such as anxiety, depression, and bipolar disorder, and removing SNPs associated with any of these potential confounders on a genome-wide basis.

### Statistical analysis

Inverse variance weighting (IVW) [13], MR-Egger regression [14], and weighted median method (WME) [15] were used for the two-sample MR analysis[16]. The results of IVW as a first priority were most reliable in the absence of heterogeneity and pleiotropy [13]. MR-Egger played an important role in detecting and correcting pleiotropy. WE considered that at least half of IVs are effective and can allow the existence of heterogeneity. Heterogeneity was tested among instrumental variables for heterogeneity, where more significant variability is associated with greater heterogeneity. Heterogeneity was evaluated by IVW and MR-Egger regression with Cochran's Q test. Instrumental variables with more significant variability were detected by the MR-MRPRESSO R package. Multiplicity tests were performed by MR Egger plot intercepts to analyze the horizontal multiplicity of instrumental variables. Suppose the intersection of the line representing the MR Egger analysis is further from the origin than the intersection of the vertical axis. In that case, the more significant the multiplicity and the instrumental variables may affect the outcome by interacting with other phenotypes. After each SNP deletion, a one-by-one elimination test was performed to verify the differences between MR and total results calculated for the remaining instrumental variables. The above analyses were analyzed and visualized in R v.4.2.0, and the relevant R packages included "MRPRESSO 1.0" and " TwoSampleMR 0.4.25" and its dependent extensions.

## Results

### Selection of instrumental variables

A total of 32 SNPs highly related to JAK2 were identified. The overall F statistics in the current study F = 72.032, and every SNP worked out as F > 10, indicating a powerful tool. Then, 29 SNPs shared by JAK2 and ED were selected as tool variables (Table 1). Specifically, rs4687657, which was associated with psychological factor, was removed from these SNPs due to association with confounders in the following study.

**Table 1:** 29 SNPs for which exposure matched with outcome

SNP	P.exposure	P.outcome	eaf.exposure	eaf.outcome	r^2^	F
rs10988220	1.514E-06	0.271	0.133	0.123	0.007	24.898
rs113218396	4.571E-06	0.147	0.058	0.058	0.007	22.180
rs116345643	1.148E-09	0.998	0.068	0.097	0.013	42.772
rs117288663	8.511E-09	0.351	0.147	0.151	0.010	32.794
rs137986280	6.761E-07	0.549	0.021	0.008	0.008	27.956
rs1460026	8.318E-10	0.662	0.801	0.773	0.012	38.466
rs1580191	1.820E-06	0.007	0.323	0.334	0.007	23.932
rs16964261	7.244E-09	0.197	0.056	0.032	0.010	34.311
rs17792426	2.291E-10	0.887	0.299	0.354	0.012	39.772
rs1801020	1.622E-11	0.418	0.750	0.738	0.014	45.476
rs2027169	1.622E-06	0.173	0.308	0.405	0.007	23.462
rs241778	2.951E-10	0.498	0.113	0.121	0.012	41.462
rs241787	1.413E-09	0.439	0.760	0.787	0.012	39.331
rs28747001	1.660E-06	0.194	0.175	0.176	0.009	29.123
rs28858474	1.995E-06	0.890	0.197	0.164	0.007	24.051
rs34517613	1.778E-06	0.844	0.137	0.079	0.007	23.797
rs3775298	5.248E-28	0.757	0.513	0.575	0.036	122.511
rs4687657	7.943E-08	0.679	0.263	0.287	0.009	29.275
rs4919420	2.239E-10	0.828	0.414	0.293	0.012	40.858
rs6809081	1.202E-20	0.807	0.423	0.513	0.027	91.648
rs704	3.467E-61	0.183	0.467	0.420	0.076	272.789
rs72840032	5.754E-14	0.600	0.047	0.039	0.018	60.016
rs74660304	3.020E-06	0.876	0.019	0.006	0.007	22.710
rs75041127	3.467E-06	0.794	0.051	0.029	0.009	30.209
rs7762757	3.890E-06	0.908	0.395	0.397	0.006	21.326
rs78419586	1.000E-08	0.468	0.082	0.041	0.010	34.292
rs79892925	3.311E-06	0.660	0.097	0.137	0.007	21.665
rs8076992	2.818E-09	0.072	0.073	0.036	0.011	35.593
rs8082039	2.138E-08	0.581	0.067	0.038	0.010	31.974

^a^*SNP* single nucleotide polymorphism

^b^*eaf* effect allele frequency: the frequency of occurrence of the effect genetic allele

### Mendelian randomization results

**Fig. 2:**
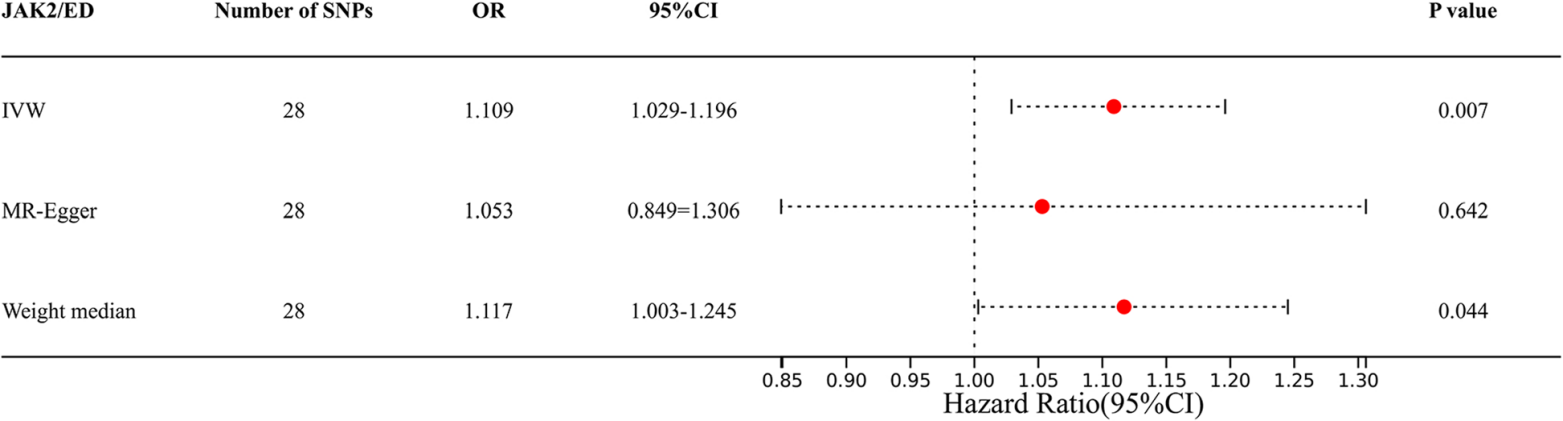
Odds ratios (ORs) for estimates of the relationship between genetically predicted JAK2 and erectile dysfunction. CI, confidence interval. The three lines represent inverse variance weighting (IVW), MR Egger, and Weight media methods, respectively

IVW showed a statistically significant potential causal effect of JAK2 on ED risk (OR = 1.109, 95% CI = 1.029–1.196, *p* = 0.007) (Fig. 2). Meanwhile, an association in the same direction was obtained using weighted median method (OR = 1.117, 95% CI = 1.003–1.245, *p* = 0.044). Considering that IVW has the advantage of maintaining higher accuracy in estimation in MR analysis compared with MR, the results of MR analysis may support the potential causal relationship between JAK2 and ED. Multivariate MR analysis revealed that there was still a significant causal relationship between JAK2 and ED under the effect of adjusting diabetes (OR = 1.084, 95% CI = 1.013–1.154, *p* = 0.026).

### Sensitivity analysis

**Fig. 3:**
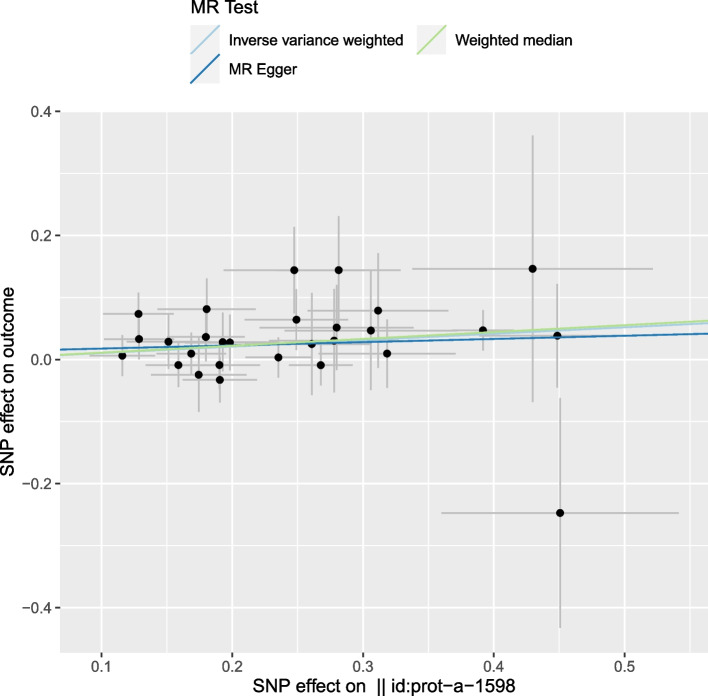
Scatter plot of the effect of SNPs on JAK2 and ED, where the different slopes of the three lines represent the estimated Mendelian randomization effect of the three MR tests. A point represents an SNP, the horizontal axis represents the effect of exposure, and the vertical axis represents the effect of outcome

**Fig. 4:**
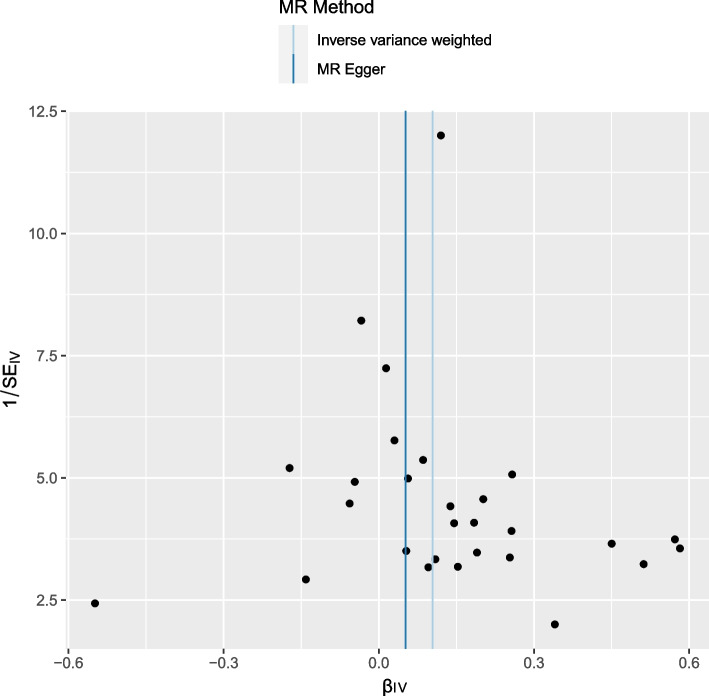
Funnel plot for instrumental variables; each point represents one SNP and a uniform distribution on both sides indicates a small heterogeneity

**Fig. 5:**
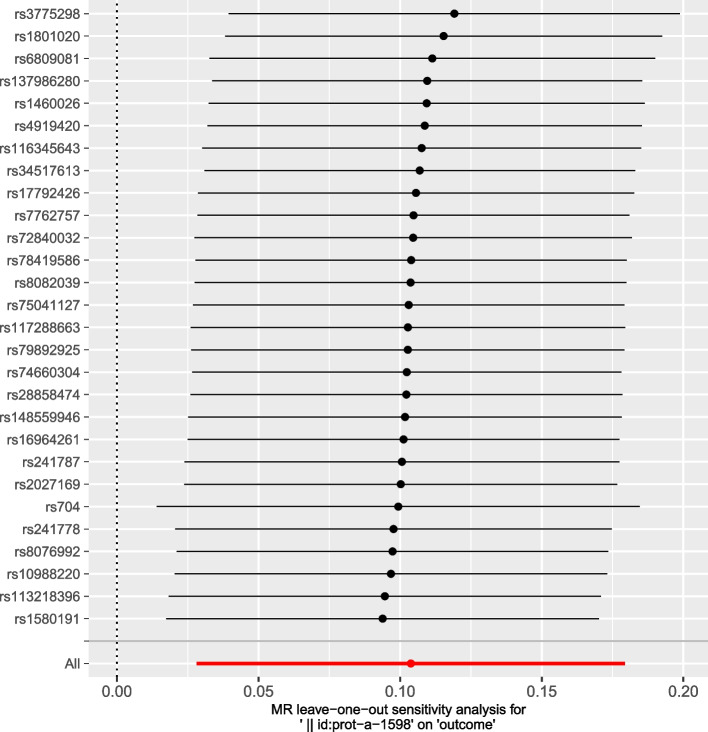
Result of forest plot for leave one out and the red bottom line represents a positive IVW result. Each line represents the result of IVW when a certain SNP is removed. The bottom red line represents the result when all SNPs are included

No heterogeneity was observed using the Cochran Q-test (IVW: *P* = 0.855; MR-Egger: *P* = 0.829). MR-PRESSO presented similar results (*P* = 0.866 in heterogeneity for the global test). Correlations between JAK2 and ED represented by the 28 SNPs were shown in Fig. 3. Moreover, the statistical significance of the intercept evidence was negative (intercept = 0.012; SE = 0.025, *p* = 0.617), indicating no pleiotropy was observed. The funnel plot was basically symmetrical, indicating that there was no heterogeneity of IVs (Fig. 4). The leave-one-out analysis indicated that the MR results were not driven by a single SNP (Fig. 5).

### Risk analysis

Since MR studies carry a certain risk of pleiotropy, we employed strategies to detect and correct potential pleiotropy. For this, we retrieved IVs related second phenotypes on the PhenoScanner to exclude the interference of confounding factors. We found that rs4687657 may be associated with some psychiatric disorders. Many studies have reported that mental factors are closely related to the occurrence of ED [1, 17], but there was no MR analysis of the relationship between mental factors and ED. We found no significant changes in the direction and statistical significance of the MR estimates before (IVW: OR = 1.108, 95% CI = 1.029–1.194, *p* = 0.007) and after the removal of rs4687657. Therefore, the results of our analysis were unlikely to be seriously affected by this pleiotropy.

## Discussion

The role of JAK2 in ED has long been of interest to scholars. However, the causal relationship between JAK2 and ED remains unclear. Previous animal and observational studies have been influenced by other factors and costs. Thus, researchers failed to clarify the causal connection. Using two-sample MR, we confirmed that JAK2 is a risk factor for ED and strengthens the evidence for a causal relationship between JAK2 and ED.

JAK2 plays an essential role in multiple biological functions. Previous studies reported that JAK2/STAT3 signaling pathway was inactivated by FNDC5, and local inflammation and oxidative stress were reduced accordingly[2]. Physiologically, nitric oxide (NO) released from parasympathetic nerve endings causes cavernous smooth muscle relaxation, which in turn causes an increase in blood flow[18, 19]. Reactive oxygen species or superoxide formed during oxidative stress reacts with NO to create peroxynitrite (ONOO-), thus reducing the concentration of NO required for the relaxation process. In contrast, the formed peroxynitrite contributes to oxidative damage to essential biomolecules[20, 21]. Accordingly, JAK2-mediated local inflammation and oxidative activation may affect the physiological processes of erection and may increase the risk of prostatitis and prostate cancer. In addition, inflammatory response is also an important response in JAK2 mediated pathway. Chengquan Ma et al. confirmed that a history of prostatitis was an independent risk factor for ED[22]. Meanwhile, chronic low-grade inflammation is essential to ED pathogenesis and a possible intermediate stage of endothelial dysfunction[23]. We suggest that JAK2 may be one of the pathways through which the inflammatory-like local inflammatory effects of the prostate exacerbate ED.

In rodent models, JAK2 was activated in the penile tissue of diabetic mice, and tamoxifen-induced JAK2 deficiency ameliorated impaired erectile function induced by diabetes, which may be mediated by a reduction in oxidative stress, apoptosis, and cavernous fibrosis[6]. The study indicated that JAK2 deficiency did not significantly affect erectile function in mice without DM. But it was unclear whether non-diabetic mice that undergo tamoxifen induction affect other indicators of the mice and cause this mere factor of JAK2 deficiency to affect non-diabetic mice in the study. Importantly, our analysis did not contradict it and is taken together. It appears to complement the studies of JAK2 concerning ED. In another study of ED pathogenesis in diabetic mice[5], oxidative stress was observed in diabetes-induced ED (DMED). The relative expression ratio of phosphorylated JAK2/JAK2 was significantly greater in the DMED group compared to the DMED group with monomeric berberine intervention. The DMED group treated with the JAK2 inhibitor AG490 improved erectile function, reduced phosphorylated JAK2, and reduced oxidative stress in the DMED mice. Although these are pilot studies based on erectile dysfunction in diabetic mice, there is still reason to believe that a corresponding pathological impairing effect on ED in JAK2-mediated oxidative stress exists.

JAK inhibitors selectively inhibit JAK kinase, block the JAK/STAT signaling pathway, and are used clinically to screen drugs for the treatment of hematological disorders, oncology, rheumatoid arthritis, and psoriasis. There is a potential role in the treatment of ED by intervening or influencing the activation of JAK2. During chemotherapy or radiotherapy for prostate cancer, damage to the cavernous nerve, a postganglionic branch of the pelvic ganglion, may lead to ED, and brain-derived neurotrophic factor (BDNF) promotes nerve regeneration by activating the JAK2/STAT pathway in Schwann cells[24, 25]. The JAK/STAT signaling pathway is also considered one of the three essential components of sponge nerve regeneration. The rest include brain-derived neurotrophic and vascular endothelial growth factors[26]. In addition, potential stem cells from different tissues may be used for erectile dysfunction recovery in animal models through local transplantation or paracrine signaling coincidentally[27], stem cells exert signaling and regenerative effects with the help of the JAK/STAT family of signaling molecules including JAK2[28]. Although no JAK inhibitors have been reported in erectile dysfunction, it is possible to inhibit the JAK/STAT pathway-mediated inflammatory response and oxidative stress through JAK inhibitors, thereby avoiding the development of erectile dysfunction. Our findings indicated that JAK2 is a risk factor for ED, suggesting that targeting JAK inhibitors might be novel and promising therapeutic candidates in the treatment of ED. Although JAK2 has not been yet reported as a genetic locus associated with ED risk in human genetic studies, we could also judge a causal relationship between JAK2 and ED risk with this new approach and further studies are required to prove the association of these genetic loci with ED.

This study has the following advantages. We addressed the causal relationship between JAK2 and ED through a Mendelian randomization study, effectively avoiding the confounding bias of random assignment of SNPs at conception. Mendelian randomization is an in-depth study based on GWAS’s extensive sample data simulating a randomized controlled trial, which is less costly and avoids reverse causal effects compared to observational studies. Moreover, our findings may guide using JAK inhibitors in ED. However, our study has some limitations. The GWAS data in our study were derived from European populations, and the extension of the results to other people needs to be further investigated. There is another ED-related GWAS data [29], but due to its earlier data year (2018 vs 2022) and shallow sequencing depth (9,310,196 vs 20,151,730) compared to the FinnGen Project, we choose to use the latest FinnGen Project results and the trend of another dataset is consistent with our results after analysis. In addition, the GWAS database does not yet specify the diversity within ED diseases, and we will consider more extensive studies on age and pathological subgroups in the future.

## Conclusions

We applied a two-sample MR to investigate the risk of JAK2 and ED in this study. Our results indicated that JAK2 was a risk factor for ED. Our study might provide an essential reference for pathological studies of ED and the use of JAK inhibitors on ED, which needed more experiments for validation.

## Data Availability

All data could be found in the publicly available online database IEU OpenGWAS Project (https://gwas.mrcieu.ac.uk/) and FinnGen project (https://r8.finngen.fi/).

## Abbreviations

JAK2: Janus kinase 2
ED: Erectile dysfunction
JAK: Janus kinase
JH2: Janus kinase homology pseudokinase
STAT: Signal transducer of activators of transcription
MR: Mendelian randomization
RCT: Randomized controlled trials
GWAS: Genome-wide association studies
SNPs: Single nucleotide polymorphisms
IVW: Inverse variance weighting
IVs: Instrumental variables
WME: Weighted median method
NO: Nitric oxide
DMED: Diabetes-induced erectile dysfunction
